# The Effect of Bonding Strategy and Aging on Adhesion to Primary Enamel: An In-Vitro Study

**DOI:** 10.3290/j.jad.b4515497

**Published:** 2023-10-16

**Authors:** Antonín Tichý, Yi Yang, Mahmoud Sayed, Yasushi Shimada, Keiichi Hosaka

**Affiliations:** a Assistant Professor, Institute of Dental Medicine, First Faculty of Medicine of the Charles University and General University Hospital in Prague, Prague, Czech Republic; Department of Cariology and Operative Dentistry, Tokyo Medical and Dental University, Tokyo, Japan. Idea, experimental design, investigation, statistical analysis, wrote the manuscript.; b PhD Candidate, Department of Cariology and Operative Dentistry, Graduate School of Medical and Dental Sciences, Tokyo Medical and Dental University, Tokyo, Japan. Investigation, proofread the manuscript, contributed substantially to discussion.; c Postdoctoral Fellow, Oral Health Science Center, Tokyo Dental College, Tokyo, Japan. Investigation, proofread the manuscript, contributed substantially to discussion.; d Professor, Department of Cariology and Operative Dentistry, Graduate School of Medical and Dental Sciences, Tokyo Medical and Dental University, Tokyo, Japan. Consulted on idea, experimental design, proofread the manuscript, contributed substantially to discussion.; e Professor, Department of Regenerative Dental Medicine, Tokushima University, Tokushima, Japan; Professor, Division of Interdisciplinary Research for Medicine and Photonics, Institute of Post LED Photonics, Tokushima University, Japan. Consulted on idea, experimental design, proofread the manuscript, contributed substantially to discussion.

**Keywords:** bond strength, durability, etch-and-rinse, self-etch, universal adhesive

## Abstract

**Purpose:**

Resin composites are commonly used in pediatric dentistry, but there is limited evidence on adhesion to primary teeth, especially primary enamel. In this study, three bonding strategies were assessed – one-step self-etch (1-SE), two-step self-etch (2-SE), and three-step etch-and-rinse (3-ER) – by measuring the immediate and aged microshear bond strength (µSBS) to sound primary enamel.

**Materials and Methods:**

120 extracted human primary molars with sound buccal surfaces were used for µSBS testing. Six adhesive protocols (two per bonding strategy) were selected and µSBS was measured either after 24 h or 10,000 thermal cycles (n = 10). Confocal laser scanning microscopy (CLSM) and scanning electron microscopy (SEM) were used to determine failure modes. Furthermore, 18 primary molars were etched using the different adhesive protocols (n = 3) for the measurement of surface roughness (Sa) using CLSM and morphological analysis using SEM.

**Results:**

After 24 h, there was no significant difference in µSBS between 1-SE and 2-SE strategies (p = 0.96), but the 2-SE strategy yielded significantly higher µSBS after thermocycling (p < 0.001). The highest µSBS was obtained using the 3-ER strategy regardless of aging (p < 0.001). The 3-ER strategy clearly exposed enamel prisms and resulted in the highest Sa (p < 0.001). In contrast, if SE strategies were used, enamel prisms were barely recognizable, and Sa was not significantly different from baseline (p > 0.95).

**Conclusion:**

The 3-ER strategy is optimal for bonding to primary enamel. The etching effect of SE strategies is weaker, resulting in lower µSBS. Thermocycling revealed that the bonding durability of the 1-SE strategy is inferior to that of to multi-step strategies.

Resin composites are increasingly used in pediatric dentistry; it is estimated that they are utilized in more than 50% of intracoronal restorations.^28^ However, limited evidence is available on adhesion to primary teeth, even though it is one of the main factors for the success of composite restorations. A large body of data has been published on permanent teeth, but this cannot be extrapolated to primary teeth, given the differences between them. Primary enamel and dentin are thinner than permanent hard dental tissues, and they are also less mineralized.^7^ Furthermore, aprismatic enamel is more pronounced in primary teeth,^13^ and since tubular density is higher in primary dentin, there is less intertubular dentin compared to permanent teeth.^16^ As a result, bond strength to primary hard dental tissues is significantly lower compared to permanent teeth, especially to dentin.^20^

Various bonding strategies are currently available, and their selection in pediatric dentistry is influenced by the fact that patients’ cooperation is often limited. Three-step etch-and-rinse (3-ER) adhesives are known to provide reliable bonding to hard dental tissues,^26^ but the application procedure is relatively time-consuming and technique sensitive. The drawbacks are similar for two-step etch-and-rinse (2-ER) adhesives, because they also require etching and rinsing in a separate step. In contrast, two-step self-etch (2-SE) and particularly one-step self-etch (1-SE) adhesives allow faster application that is less prone to errors. On the other hand, contemporary SE adhesives are mildly acidic, so their ability to etch enamel is substantially lower compared to phosphoric acid.^27^ In addition, 1-SE adhesives lack the non-solvated hydrophobic bonding layer, thus being more susceptible to water sorption and hydrolytic degradation.^23^

In pediatric dentistry, adhesive strategies have been evaluated in few clinical trials,^9^ so the decision-making process is usually based on in-vitro studies. Their meta-analysis published in 2016 showed that the highest pooled immediate bond strength was obtained with 2-SE and 2-ER adhesives on dentin and with 3-ER adhesives on enamel. In contrast, 1-SE adhesives exhibited the lowest immediate bond strength on both substrates.^15^ However, these findings must be interpreted with caution, because clinical outcomes correlate better with aged bond strength.^8^ In the meta-analysis, 3-ER adhesives had the highest aged bond strength on dentin, but this was only based on four studies, and none of the included studies tested aged bond strength to enamel.^15^

Since then, in-vitro studies have focused mainly on the latest generation of adhesives, labeled as universal or multi-mode, as they can be applied either in the SE or ER mode. However, the lack of studies on adhesion to primary enamel persists to date, which was confirmed by two recently published systematic reviews and meta-analyses.^6,10^ Although we found three studies that performed some aging procedures^2,11,12^ and were not included in the aforementioned meta-analyses, the number of thermal cycles was low (≤ 5000) and there was no comparison with immediate bond strength that would indicate the effect of aging. Therefore, the objective of this study was to assess various bonding strategies (1-SE, 2-SE, 3-ER) by measuring the immediate and aged microshear bond strength (µSBS) to sound human primary enamel. In addition, the etching effect of the bonding strategies was analyzed by measuring the enamel surface roughness using confocal laser scanning microscopy (CLSM). The null hypotheses were that the bond strength of the tested adhesives would not be affected by 1) the bonding strategy and 2) aging procedure (10,000 thermal cycles).

## Materials and Methods

### Ethical Considerations

In total, 138 extracted carious primary molars with a sound buccal surface were used in this study – 120 for µSBS testing and 18 for morphological analyses of the etched surfaces. The use of extracted primary teeth was approved by the Ethics Committee of the General University Hospital in Prague, protocol number 101/21 S-IV. Written informed consent for the extraction and use of the teeth for research purposes was obtained from the children’s parents.

### Processing of the Extracted Teeth

**Fig 1:**
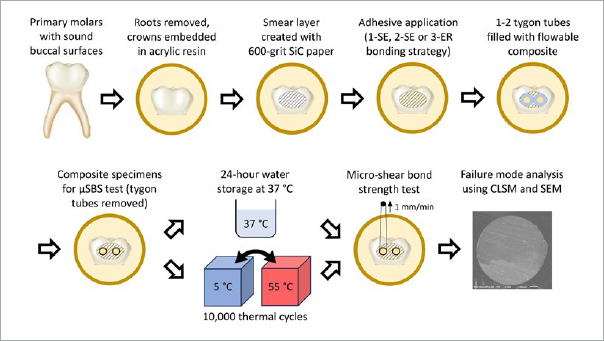
Schematic illustration of the µSBS test.

**Fig 2:**
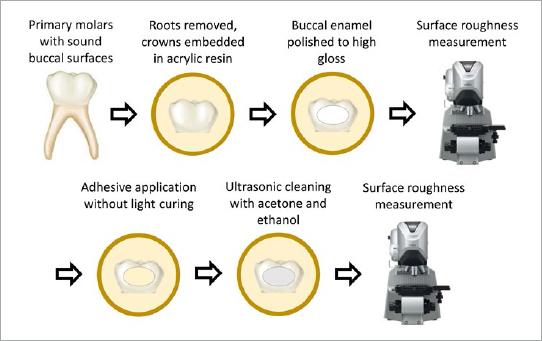
Schematic illustration of the surface roughness measurement.

After extraction, any soft tissues were removed from the surface and the teeth were placed in a 0.5% chloramine-T solution for 1 week. The teeth were then stored at 4°C in tap water, which was changed weekly for up to three months. Prior to the experiments, roots of the teeth were removed using a model trimmer, and the crowns were embedded in a clear self-curing acrylic resin (Unifast II, GC; Tokyo, Japan), leaving buccal surfaces exposed. For µSBS testing (Fig 1), the buccal surfaces were flattened using a 600-grit SiC paper (DCCS, Sankyo Fuji Star; Saitama, Japan) under running water to produce a standardized smear layer. For the measurements of surface roughness (Fig 2), the buccal surfaces were polished to high gloss using 2000-grit SiC papers (DCCS, Sankyo Fuji Star) under running water and diamond polishing pastes (6 µm, 3 µm, 1 µm and 0.25 µm; DP-Paste P, Struers; Copenhagen, Denmark). The buccal surfaces were then rinsed with water and gently air dried.

### Adhesive Application

The tested adhesives included two one-bottle universal adhesives, G-Premio Bond (GPB, GC) and Clearfil Universal Bond Quick ER (UBQ, Kuraray Noritake; Tokyo, Japan), a two-bottle universal adhesive, G2 Bond Universal (G2U, GC), and a 2-SE adhesive, Clearfil SE Bond 2 (CSE2; Kuraray Noritake Dental). A K-etchant syringe (Kuraray Noritake) was used for enamel etching in the 3-ER strategy.

The teeth were randomly divided into six groups according to the adhesive application protocol, and each group was further subdivided according to the aging procedure (n = 10), i.e., 24-h water storage and thermocycling. GPB and UBQ were applied in the SE mode (1-SE strategy), G2U and CSE2 were applied either in the SE mode (2-SE strategy) or after the enamel surface had been etched with K-etchant for 10 s and rinsed with water for 10 s (3-ER strategy). The adhesives were applied according to the respective manufacturer’s instructions (Table 1). Light curing was performed using a Valo LED polymerization lamp (Ultradent; South Jordan, UT, USA) in the standard mode (1000 mW/cm^2^) from a distance of approximately 2 mm.

**Table 1:** Composition, batch number, and application procedure of the tested adhesives

Material (abbreviation; manufacturer)	Composition (batch number)	Application procedure
G-Premio Bond (GPB, GC; Tokyo, Japan)	4-MET, MDP, MDTP, methacrylate monomer, acetone, water, TPO, silica (2012031)	1. Apply to the enamel surface and leave for 10 s. 2. Air dry strongly for 5 s. 3. Light cure for 10 s.
Clearfil Universal Bond Quick ER (UBQ, Kuraray Noritake; Tokyo, Japan)	MDP, bis-GMA, HEMA, hydrophilic amide monomer, colloidal silica, ethanol, water, silane coupling agent, sodium fluoride, CQ (CJ0111)	1. Apply to the enamel surface. 2. Air dry immediately for 5 s with mild air pressure. 3. Light cure for 10 s.
G2 Bond Universal (G2U, GC)	Primer: 4‐MET, MDP, MDTP, dimethacrylates, water, acetone, photoinitiator, filler (2010051) Bonding agent: dimethacrylates, filler, photoinitiator (2010131)	(0. ER-mode: Apply etchant to the enamel surface, leave for 10 s, rinse with water for 10 s, air dry for 5 s.) 1. Apply primer to the enamel surface and leave for 10 s. 2. Air dry strongly for 5 s. 3. Apply bonding agent, air blow gently for 3 s. 4. Light cure for 10 s.
Clearfil SE Bond 2 (CSE2, Kuraray Noritake)	Primer: MDP, HEMA, Hydrophilic aliphatic dimethacrylate, water, CQ (450089) Bonding agent: MDP, bis-GMA, HEMA, Hydrophobic aliphatic dimethacrylate, CQ, initiators, accelerators, silica (5N0140)	(0. ER-mode: Apply etchant to the enamel surface, leave for 10 s, rinse with water for 10 s, air dry for 5 s.) 1. Apply primer to the enamel surface and leave for 20 s. 2. Air dry for 5 s with mild air pressure. 3. Apply bonding agent, air blow gently for 3 s. 4. Light cure for 10 s.

Abbreviations: 4-MET: 4-methacryloxyethyl trimellitic acid; MDP: 10-methacryloyloxydecyl dihydrogen phosphate; MDTP: 10-methacryloyloxydecyl dihydrogen thiophosphate; TPO: diphenyl(2;4;6-trimethylbenzoyl)phosphine oxide; bis-GMA: bisphenol-A-glycidyl methacrylate; HEMA: 2-hydroxyethyl methacrylate; CQ: camphorquinone; ER: etch-and-rinse.

### Specimen Preparation

Tygon tubes (Saint Gobain Performance Plastic; Paris, France) with a 0.79-mm internal diameter and 1.0 mm height were placed onto the bonded enamel surface. While stabilized using tweezers, the tubes were filled with a flowable resin composite (Estelite Universal Flow Medium, shade A2, Tokuyama Dental; Tokyo, Japan) and light cured for 20 s using the Valo lamp. Five minutes after polymerization, the wall of each tube was cut using a razor blade and removed. Despite careful handling, some specimens detached from the surface with the tygon tube, but they were not considered pre-testing failures, because their detachment was caused by the edge of the razor blade and/or the presence of a bubble near the base of the composite cylinder. The number of specimens per group was 13-16, because one or two tygon tubes could be placed on the bonded enamel surface, depending on its area.

### Aging and Microshear Bond Strength Test

After 24 h of storage in distilled water at 37°C, half of the teeth were subjected to 10,000 thermal cycles between 5°C and 55°C with a dwell time of 30 s and a transfer time of 2 s. The teeth were then attached to a testing jig in the universal testing machine (EZ Test, Shimadzu; Kyoto, Japan), a loop of 0.2-mm wire was aligned with the resin-enamel interface,^19^ and the bonded specimens were subjected to the µSBS test at a crosshead speed of 1 mm/min.

### Failure Mode Analysis

After the µSBS test, the enamel surfaces were desiccated in a desiccator for 24 h, and failure modes were classified using a confocal laser scanning microscope (CLSM, VK-X150, Keyence; Osaka, Japan) at 100X magnification. Three failure modes were distinguished: 1. adhesive failure (more than 80% of the failure at the enamel-adhesive interface or within the adhesive layer); 2. cohesive failure (more than 80% of the failure in the resin composite); 3. mixed failure (if neither type 1 nor 2 were present on at least 80% of the fracture surface). The specimens were then sputter-coated with gold and observed using a scanning electron microscope (SEM; JSM-IT100, JEOL; Tokyo, Japan) to confirm the classification and to verify that the bonded surface was only enamel.

### Surface Roughness Measurement and Morphological Analysis of Etched Enamel Surfaces

The teeth were randomly divided into six groups (n = 3) according to the adhesive application protocols. The polished enamel surfaces were thoroughly air dried, and their roughness was measured at 4 locations per tooth using the CLSM at 1000X magnification. The surfaces were then rinsed with water, gently air dried, and the adhesives were applied as described above for the µSBS test. However, the adhesives were not light cured; instead, the surfaces were ultrasonically cleaned with acetone and ethanol (5 min each) to remove the adhesive from the enamel surface. The specimens were then air dried, and surface roughness was measured again. In total, there were 12 measurements before and after adhesive application per group. The MultiFileAnalyzer software (version 1.3.1.120, Keyence) was used to process the data. Finally, the enamel surfaces were desiccated in a desiccator for 24 h, sputter-coated with gold, and the etching patterns were observed using SEM at 5000X magnification.

### Statistical Analyses

The analyses were performed using Statistica software (version 14.0, TIBCO Software; Palo Alto, CA, USA), and the significance level was set to 0.05. The µSBS data were statistically analyzed using a two-way ANOVA (independent variables: adhesive, aging condition) followed by Tukey’s post-hoc test. To assess the effect of the bonding strategy, another two-way ANOVA (independent variables: bonding strategy, aging condition) with Tukey’s post-hoc test was performed. In the analyses, the single pre-test failure assigned a value of the mean between 0 MPa and the µSBS in the specific experimental group.^3^

Surface roughness was evaluated using the Sa parameter that expresses the difference in height of each point relative to the arithmetical mean of the surface. The four values measured on each surface were averaged, and the mean values at baseline and adhesive application (n = 3) were processed statistically. A two-way repeated measures ANOVA (independent variables: adhesive, aging condition) followed by Tukey’s post-hoc test was used to compare the etching effect of individual adhesives, and another two-way repeated measures ANOVA (independent variables: bonding strategy, aging condition) was performed to compare the 1-SE, 2-SE, and 3-ER strategies.

## Results

### Bond Strength

The two-way ANOVA revealed that bonding strategy had a significant effect on µSBS (p < 0.001). Aging condition was not a significant factor (p = 0.63), but its interaction with bonding strategy was significant (p = 0.001). Tukey’s post-hoc tests revealed no significant difference between 1-SE and 2-SE strategies after 24 h (p = 0.96), but the µSBS of the 2-SE strategy was significantly higher after thermocycling (p < 0.001). The highest µSBS was obtained using the 3-ER strategy, regardless of the aging condition (p < 0.001). The effect of thermocycling was not significant for any of the strategies. However, while a slight increase in µSBS was observed in 2-SE and 3-ER strategies after thermocycling (p = 0.80 and p = 0.16, respectively), the µSBS of the 1-SE group decreased (p = 0.07). Mean µSBS and significant differences between individual adhesives are presented in Table 2, which also presents the failure mode distributions.

**Table 2:** Microshear bond strength (mean ± SD) and failure mode

Strategy	Adhesive	24 h		Thermocycling	
		µSBS (MPa)	Failure mode (A-M-C)	µSBS (MPa)	Failure mode (A-M-C)
1-SE	GPB	22.3 ± 10.2^AB^	9-3-1	18.8 ± 4.5^AB^	11-2-0
	UBQ	20.0 ± 7.1^AB^	12-1-0	12.8 ± 6.7^A^	12-1-0
2-SE	G2U	22.2 ± 6.0^AB^	7-6-0	26.1 ± 7.9^B^	11-3-0
	CSE2	23.3 ± 4.0^AB^	11-3-0	24.4 ± 5.8^B^	12-1-0
3-ER	G2U-ER	32.6 ± 6.8^C^	7-6-0	35.5 ± 6.6^C^	8-5-0
	CSE2-ER	29.4 ± 8.9^BC^	15-0-0	35.2 ± 7.4^C^	11-4-1

Different superscript letters indicate a significant difference (p < 0.05) between groups in each column. There was no significant difference between µSBS after 24 h and thermocycling for any of the adhesives (p > 0.05). SE: self-etch; ER: etch-and-rinse; GPB: G-Premio Bond; UBQ: Clearfil Universal Bond Quick; G2U: G2-Bond Universal; CSE2: Clearfil SE Bond 2; A: adhesive failure; M: mixed failure; C: cohesive failure in resin composite.

### Failure Mode

**Fig 3:**
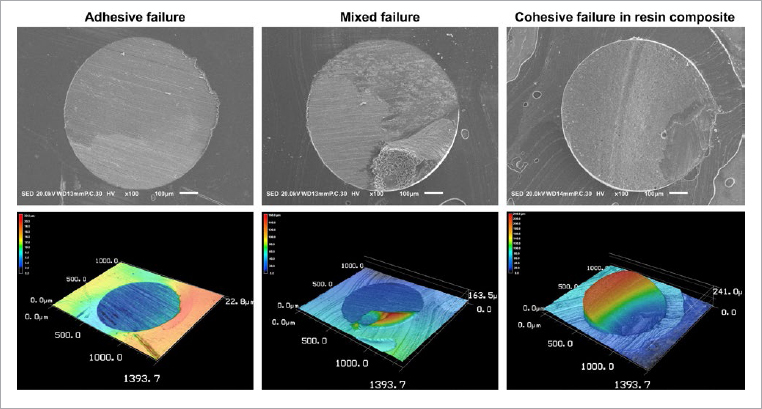
Representative images of failed specimens. The first row displays scanning electron micrographs at a magnification of 100X. The second row presents the same surfaces observed using the confocal laser scanning microscope.

Overall, adhesive failures prevailed both after 24 h (75%) and after thermocycling (79%). The incidence of mixed failures was 24% after 24 h and 20% after thermocycling. There were just two cohesive failures in the resin composite, one in the GPB group after 24 h and the other in the CSE2-ER group after thermocycling. There was one pre-test failure in the GPB group after 24 h. Representative SEM and CLSM images of the failure modes are presented in Fig 3.

### Surface Roughness

Statistical analysis disclosed no significant difference between the Sa of 1-SE and 2-SE strategies (p = 0.98), but it revealed the surface roughness to be significantly higher in the 3-ER strategy (p < 0.001). The difference between baseline and the etched state was also significant only for the 3-ER groups (p < 0.001), in which Sa increased more than threefold. In the SE groups, the increase in Sa was approximately 55% for GPB and G2U, while there was almost no difference from baseline for UBQ and CSE2 (Table 3).

**Table 3:** Surface roughness (Sa) in µm, mean ± SD

Strategy	Adhesive	Baseline	Etched	Difference after etching
1-SE	GPB	0.080 ± 0.003^A^	0.124 ± 0.035^A^	+ 0.044 (+ 55%)
	UBQ	0.141 ± 0.025^A^	0.143 ± 0.011^A^	+ 0.002 (+ 2%)
2-SE	G2U	0.087 ± 0.021^A^	0.135 ± 0.053^A^	+ 0.048 (+ 55%)
	SE2	0.096 ± 0.031^A^	0.099 ± 0.029^A^	+ 0.003 (+ 4%)
3-ER	G2U-ER	0.097 ± 0.016^A^*	0.518 ± 0.010^B^*	+ 0.421 (+ 434%)
	SE2-ER	0.108 ± 0.006^A^*	0.474 ± 0.145^B^*	+ 0.366 (+ 337%)

Different superscript letters indicate a significant difference (p < 0.05) between groups in each column. *Significant difference (p < 0.05) between baseline and the etched state. SE: self-etch, ER: etch-and-rinse, GPB: G-Premio Bond, UBQ: Clearfil Universal Bond Quick, G2U: G2-Bond Universal, CSE2: Clearfil SE Bond 2.

### Morphological Analysis of Etched Enamel Surfaces

**Fig 4:**
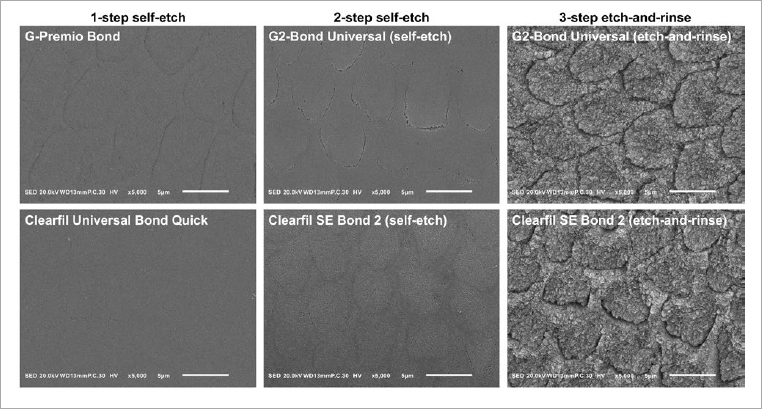
Scanning electron micrographs of etched enamel surfaces at a magnification of 5000X.

The SEM analysis of the etched surfaces confirmed the results of the surface roughness measurements. The outlines of enamel prisms were barely recognizable on the surfaces treated with UBQ and just slightly more marked for other SE adhesives. In contrast, in the 3-ER strategy, phosphoric acid etching resulted in deeper demineralization (Fig 4). Demineralization was most pronounced at the periphery of the prisms, as opposed to prism cores, which were less affected. This corresponds to the Silverstone type-II etching pattern.^21^

## Discussion

As amalgam is gradually phased down, composite restorations are increasingly used in pediatric dentistry. However, while many studies evaluating adhesion to permanent teeth are available, the evidence for primary teeth is scarce.^6,10,15^ In particular, very few studies have assessed the bonding to primary enamel and its durability. Therefore, this study evaluated three bonding strategies and their performance on sound primary enamel after 24 h and 10,000 thermal cycles. The results led to the rejection of the first null hypothesis that the bond strength would not be affected by the bonding strategy, because the 1-SE strategy was outperformed by the 2-SE strategy after thermocycling, and because the 3-ER strategy yielded significantly higher µSBS than the SE strategies regardless of aging. The second null hypothesis could not be rejected, as the effect of thermocycling on µSBS was not significant. However, even though the decrease in the µSBS of the 1-SE strategy was non-significant, it resulted in a significant difference in aged bond strength between 1-SE and multi-step strategies.

In this study, two one-bottle universal adhesives were used in the SE mode to represent the 1-SE bonding strategy. The one-step application is straightforward and reduces time required for filling placement, which is very convenient in pediatric dentistry. Furthermore, a recent systematic review and meta-analysis concluded that “a mild universal adhesive can substitute ER and SE systems for restoring primary teeth”,^6^ suggesting that the bonding performance of universal adhesives is comparable to multi-step systems. However, only two of the included studies evaluated adhesion to sound primary enamel and neither of them tested aged bond strength,^2,4^ which is known to have a better correlation with clinical outcomes.^8^ While our immediate µSBS data support the finding of Antoniazzi et al^2^ that there was no significant difference in µSBS between 1-SE and 2-SE strategies after 24 h (2), the µSBS of the 1-SE adhesives was significantly lower than that of 2-SE adhesives after 10,000 thermal cycles (Table 2). This contradicts the conclusion of the aforementioned systematic review and meta-analysis^6^ and demonstrates that one-bottle universal adhesives used in the SE mode cannot replace multi-step systems in bonding to sound primary enamel.

The lower µSBS of 1-SE adhesives after thermocycling agrees with the findings in permanent teeth. The lower durability of 1-SE adhesives has been attributed to the increased water sorption into their adhesive layer,^5^ which contains hydrophilic monomers such as HEMA (2-hydroxyethyl methacrylate) and remnants of water and/or volatile solvents.^27^ The recently introduced one-bottle universal adhesives indeed aim at reducing the hydrophilicity – in UBQ, HEMA is partially substituted with a methacrylamide monomer,^14^ and GPB is completely free of HEMA.^22^ However, its absence has been shown to result in phase separation, i.e., the formation of water droplets within the adhesive layer.^25^ These adhesives also feature novel initiators of polymerization,^22^ but the results of this study indicate that their performance has not reached the level of multi-step adhesives to date.

The primers of 2-SE adhesives have a composition similar to that of 1-SE adhesives (Table 1), and the application of a hydrophobic bonding agent in the second step seals the adhesive interface. The second step was previously reported to improve the longevity of the adhesive joint,^1,23^ which was confirmed in this study, because the µSBS of the 2-SE strategy was not significantly affected by thermocycling. However, the contemporary 1-SE and 2-SE adhesives share the drawback of a weaker etching effect caused by their mild acidity.^27^ The statistical analysis did not show any significant difference in surface roughness between 1-SE and 2-SE strategies, but the application of GPB and G2U increased surface roughness from baseline more than UBQ and CSE2 did. This may be caused by the higher acidity of GPB and the primer of G2U (pH approximately 1.5) compared to UBQ and the primer of CSE2 (pH approximately 2.3).^22,24^

Both the SE strategies resulted in significantly lower surface roughness than the 3-ER strategy, in which enamel prims were clearly exposed (Fig 4). The more intensive etching effect of phosphoric acid enabled stronger micromechanical interlocking,^18^ leading to significantly higher µSBS using the 3-ER strategy, both after 24 h and thermocycling. Since the 3-ER strategy was tested using the 2-SE adhesives with previous phosphoric acid etching, the higher bond strength was clearly caused by the stronger demineralization. On the one hand, this result corroborates the conclusion of the meta-analysis by Lenzi et al^15^ that the in-vitro performance of ER adhesives in primary teeth is superior to SE adhesives, and it also agrees with the clinical observation of better marginal adaptation of ER adhesives by Donmez et al.^9^ On the other hand, a clinical trial by Lenzi et al^15^ revealed that phosphoric acid etching of primary molars after selective carious tissue removal prior to bonding with universal adhesives tended to decrease the survival rate of composite restorations.^17^ Since another clinical trial revealed that shorter dentin etching time tended to improve clinical outcomes in moderate occlusal lesions, further studies are required to determine the optimal bonding strategy for primary teeth. However, when interpreting the results, it should be taken into account that in-vitro studies cannot fully simulate clinical conditions. This limitation may be particularly marked in primary teeth, as the limited compliance of children may adversely affect the clinical performance of adhesives.

## Conclusion

The 3-ER bonding strategy provided the highest bond strength to sound primary enamel. The adhesion of 2-SE adhesives was weaker, but it was also stable after thermocycling, as opposed to the 1-SE strategy which resulted in inferior aged bond strength. Therefore, within the limitations of this in-vitro study, it was concluded that one-bottle universal adhesives used without phosphoric acid etching cannot completely replace multi-step systems in bonding to sound primary enamel.

## Clinical relevance:

Phosphoric acid etching is essential for strong and durable adhesion to primary enamel. Self-etch adhesives have a weak etching effect, resulting in lower bond strength. The bonding durability of universal adhesives used in self-etch mode is inferior to multi-step systems.
